# Green Tea Epigallocatechin-3-gallate (EGCG) Targeting Protein Misfolding in Drug Discovery for Neurodegenerative Diseases

**DOI:** 10.3390/biom11050767

**Published:** 2021-05-20

**Authors:** Priscila Baltazar Gonçalves, Ana Carolina Rennó Sodero, Yraima Cordeiro

**Affiliations:** Faculty of Pharmacy, Federal University of Rio de Janeiro, Rio de Janeiro 21949-900, Brazil; priscilabaltazar@live.com (P.B.G.); acrsodero@pharma.ufrj.br (A.C.R.S.)

**Keywords:** natural products, epigallocatechin-3-gallate, catechins, neuroprotective, anti-neurodegenerative, anti-amyloidogenic, misfolded proteins, amyloid-β, α-synuclein, Alzheimer’s disease, Parkinson’s disease

## Abstract

The potential to treat neurodegenerative diseases (NDs) of the major bioactive compound of green tea, epigallocatechin-3-gallate (EGCG), is well documented. Numerous findings now suggest that EGCG targets protein misfolding and aggregation, a common cause and pathological mechanism in many NDs. Several studies have shown that EGCG interacts with misfolded proteins such as amyloid beta-peptide (Aβ), linked to Alzheimer’s disease (AD), and α-synuclein, linked to Parkinson’s disease (PD). To date, NDs constitute a serious public health problem, causing a financial burden for health care systems worldwide. Although current treatments provide symptomatic relief, they do not stop or even slow the progression of these devastating disorders. Therefore, there is an urgent need to develop effective drugs for these incurable ailments. It is expected that targeting protein misfolding can serve as a therapeutic strategy for many NDs since protein misfolding is a common cause of neurodegeneration. In this context, EGCG may offer great potential opportunities in drug discovery for NDs. Therefore, this review critically discusses the role of EGCG in NDs drug discovery and provides updated information on the scientific evidence that EGCG can potentially be used to treat many of these fatal brain disorders.

## 1. Introduction

Neurodegenerative diseases (NDs) are a global public health threat and a huge financial burden for health care systems, not to mention a major hardship for society and families [1,2,3]. No effective treatment currently exists for NDs, and current therapies merely alleviate the symptoms. Thus, there is an urgent need for new, safer, and more effective drugs [4,5]. Natural products (NPs) and their unique polypharmacology provide significant advantages for drug discovery, particularly for the treatment of multifactorial and complex NDs [6,7,8,9].

The natural compound epigallocatechin-3-gallate (EGCG) has been extensively explored and studied for its therapeutic potential for NDs [10,11]. EGCG is a dietary polyphenol found in green tea with potent antioxidant and anti-inflammatory effects and an ability to modulate multiple targets implicated in the pathogenesis of many chronic diseases, including cancer, cardiovascular diseases, diabetes, and NDs [12]. 

**Figure 1:**
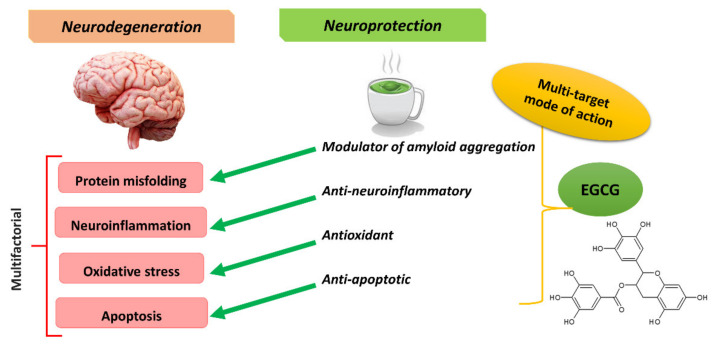
Schematic showing the role of epigallocatechin-3-gallate (EGCG) in neuroprotection. Neurodegeneration is a multifactorial process, and preclinical models have shown that EGCG exerts neuroprotective effects through several mechanisms.

The neuroprotective effects of EGCG have been reported for several NDs, including Alzheimer’s disease (AD) and Parkinson’s disease (PD), the two most common NDs [10,13]. The pathogenesis of NDs shares many fundamental processes associated with progressive neuronal dysfunction and death, with protein misfolding, oxidative stress, apoptosis, and neuroinflammation as some of them [14,15]. Meanwhile, EGCG has a multi-target mode of action (Figure 1) and synergistically acts against protein misfolding, oxidative stress, apoptosis, and neuroinflammation. Indeed, it is now recognized that the role of EGCG in ND management can be attributed to its antioxidant, anti-inflammatory, anti-apoptotic, and anti-amyloidogenic properties [16].

The pathological hallmark of many NDs is the accumulation of misfolded protein aggregates in the brain [17,18]. A growing body of evidence suggests that targeting protein misfolding is a promising strategy to prevent NDs. Protein misfolding and aggregation are principal causative factors in neurodegeneration, making their modulation a feasible target for ND prevention [17,19,20].

Protein misfolding and the consequent self-association into toxic oligomers and amyloid deposits are now considered central elements of the etiology of a wide range of NDs, including highly prevalent ones such as AD and PD, as well as rarer disorders such as prion diseases [21,22,23]. Although each ND is associated with abnormalities in the folding of a different protein, the molecular pathways leading to misfolding and aggregation appear to be similar. These findings suggest that a common therapy for NDs might be possible [24]. 

Studies have revealed that EGCG can interact with a variety of proteins linked to protein misfolding, such as Aβ, tau, α-synuclein (α-syn), transthyretin (TTR), and huntingtin [25,26,27,28]. This review addresses the contribution of EGCG research toward advances in neurodegenerative disease drug discovery. We begin by summarizing the available data of in-cell and animal ND models that support the therapeutic role of EGCG. We then focus on the protein misfolding as one of the most promising targets for ND treatment using EGCG.

## 2. Neurodegenerative Diseases

NDs are among the most widespread health problems, affecting millions of people worldwide [29]. Moreover, the number of individuals living with NDs such as AD and PD is increasing, negatively affecting families, communities, and healthcare systems worldwide [1,2]. These disorders are becoming highly prevalent, in part due to global increases in human life expectancy, since NDs are age-dependent disorders [30,31]. Indeed, aging is the primary risk factor for most NDs [32]. The impact of these diseases will further increase in the coming decades as humans live longer lives [31].

NDs are common chronic diseases typically characterized by a progressive loss of function and death of neurons in the brain or peripheral nervous system [15]. Although NDs differ in their clinical presentation, they do share several common pathological mechanisms, which are characterized by multiple targets. The underlying pathobiological processes are largely shared, with most involving the formation of abnormal protein deposits at their onset and all exhibiting a common and characteristic pattern of neuronal degeneration in anatomically or functionally related regions [14]. This idea that diverse NDs have a common cause and pathological mechanism supports that a common therapeutic strategy for these devastating disorders might be possible [24].

AD, PD, amyotrophic lateral sclerosis (ALS), and Huntington’s disease (HD) are just a few examples of NDs that share similar biochemical reactions that lead to neurodegeneration [17,29,33]. Extensive evidence shows that misfolded proteins such as Aβ and tau in AD, α-syn in PD, and TAR DNA-binding protein 43 (TDP-43) in ALS participate in the formation, accumulation, and deposition of toxic misfolded aggregates [34,35,36]. Furthermore, protein misfolding (Table 1) is one of the principal causes of the onset and progression of NDs [17,21].

It has also been proposed that the overproduction of reactive oxygen species may have a complex role in promoting disease development [38,39]. In such cases, neurodegeneration results from the excessive production of free radicals induced by fragments of insoluble and/or overproduced misfolded proteins due to functional alterations in the mitochondria, inadequate energy supply, production of inflammatory mediators, and alteration of antioxidant defenses. Oxidative stress is, therefore, considered to be a common key player in the etiology and progression of these NDs [39,40].

Protein misfolding events can promote an excessive immune response causing neuroinflammation, which is also a common feature of NDs [41]. It is hypothesized that release of protein aggregates from neurons activates microglia triggering an inflammatory response characterized by liberation of inflammatory mediators, which contribute to disease progression and severity. For instance, in AD, the glial activation is followed by nuclear factor NF-kβ activation, synthesis, and release of proinflammatory cytokines including tumor necrosis factor (TNF)-α, interleukin (IL)-1, IL-6, and IL-12 that affect neuronal receptors with an overactivation of protein kinases [41].

Neurodegeneration can, therefore, be seen as a consequence of several detrimental processes, including protein aggregation, oxidative stress, and neuroinflammation, which finally lead to the loss of neuronal functions and cognitive impairments [17,39,41]. Since NDs are multifactorial diseases related to complex pathophysiological characteristics and complicated interactions with a large number of genes and proteins, there is still no effective drug treatment of these conditions [42,43]. 

It is unlikely that targeting a single change will be effective at treating neurodegeneration, as several changes occur in the development of NDs. Given this multifactorial profile, NDs require a multi-target therapeutic approach, and current research is exploring multitarget drugs that can address more than a single event at the same time [44,45,46,47]. 

### 2.1. Alzheimer’s Disease

AD is the most common ND worldwide and also the most common cause of dementia in elderly patients [2,3]. In 2019, AD and other forms of dementia were ranked by the World Health Organization (WHO) as the seventh most common cause of death in the world [48]. To date, only five drugs (tacrine, donepezil, rivastigmine, galantamine, and memantine) have been approved by the FDA to treat AD. The disease is currently incurable, with the available drugs only managing the symptoms and exhibiting severe side effects [42,49,50]. These drugs are based on a single-target strategy and focus on restoring neurotransmitter homeostasis. Finding disease-modifying AD therapies remains an urgent and unmet clinical need [5,51].

Since the approval of memantine in 2003, the first drug approved for AD was a marine-derived oligosaccharide, sodium oligomannate. With mode of action related to gut microbiota and neuroinflammation, this latter compound was approved in 2019 in China for treating mild to moderate AD to improve cognition function [52,53].

AD is a multifactorial disease characterized by the progressive accumulation of Aβ fibrils and abnormal tau proteins in extracellular spaces and in neurons, respectively, with associated neuron and synapse loss in multiple brain regions, especially in the frontal cortex and hippocampus [54,55]. The Aβ (Aβ_40_ and Aβ_42_ with 40 and 42 amino acids) and tau proteins (352 to 421 amino acids) have been clearly identified as the key misfolded proteins in AD [56,57]. 

At the microscopic level, the brains of AD patients are characterized by the concurrent presence of two classes of abnormal structures: extracellular amyloid plaques and intraneuronal neurofibrillary tangles (NFTs). Both structures are made of highly insoluble, densely packed filaments. These structures are formed by distinct soluble building blocks: Aβ peptides for plaques and tau for NFTs [56]. 

The senile plaques and NFTs are recognized as the two major neuropathological hallmarks of AD [58,59,60]. As the major component of senile plaques, the Aβ peptide is considered to be a crucial factor that underlies neuronal and synaptic dysfunction in AD progression [61]. Hence, the amyloid hypothesis proposes Aβ as the principal cause of AD, suggesting that clinical symptoms such as memory loss and cognitive decline are caused by misfolding of the extracellular Aβ protein accumulates in senile plaques and also by intracellular deposition of misfolded tau protein [61,62,63].

In this context, Aβ has emerged as a promising therapeutic target in attempts to develop a disease-modifying treatment for AD. Most drugs tested for AD in the past 20 years have targeted the accumulation of the Aβ with a focus on decreasing levels of Aβ monomers, oligomers, aggregates, and plaques using compounds that decrease production, antagonize aggregation, or increase brain clearance of Aβ, such as β-site amyloid precursor protein cleaving enzyme 1 (BACE-1) inhibitors, and anti-Aβ antibodies [64,65,66]. Despite the large number of anti-Aβ drugs entering clinical development and the enormous expenditure on large and complex trials, these drug candidates have so far failed to show clinical benefits for AD [65]. 

It has, therefore, been suggested that the failure of drug discovery in AD reflects an incomplete understanding of disease mechanisms. Since Aβ has a physiological role, some anti-Aβ drugs that inhibit the production of ‘nascent’ Aβ, such as γ- secretase and BACE inhibitors, have been found to accelerate cognitive decline, possibly owing to off-target effects. It is speculated that more favorable outcomes might be achieved by targeting Aβ oligomers, the most neurotoxic molecular species, with encouraging results from monoclonal antibodies directed against these oligomers [66,67,68,69].

Notably, there has been a shift from an initial focus on amyloid plaques to a more contemporary view that memory failure in AD is caused by small soluble Aß oligomers acting as synaptotoxins, leading to cognitive impairment [70]. According to recent findings, Aβ oligomers play a key role in AD brain inflammation by activating the pro-inflammatory interleukin-1 (IL-1) receptors that mediate the alteration in levels of mitochondrial fission/fusion proteins, resulting in memory impairment [71].

### 2.2. Parkinson’s Disease

PD is s considered to be the second most frequent ND in the world after AD [1,72]. It has been estimated that the global burden of PD more than doubled globally from 1990 to 2015 as a result of an aging population, with potential contributions from longer disease duration and environmental factors [1]. Studies in the area have implicated environmental and genetic risk factors in the pathogenesis of PD [73]. The environmental risk factors include pesticides and ambient air pollution [74,75,76,77].

PD is a progressive ND characterized by the selective loss of dopaminergic neurons in the substantia nigra pars compacta, located in the basal ganglia of the brain, resulting in the lack of dopamine in this organ [78,79,80]. Dopaminergic cell loss causes clinical signs and symptoms such as bradykinesia, rigidity, postural instability, and tremors [73]. 

So far, no cure has been available to treat PD, with pharmacological treatments mainly consisting of dopaminergic drugs, which are only therapies to reduce symptoms that are still limited by several side effects [81]. The majority of current drugs were approved for clinical use in the second half of the twentieth century, with the development of new drugs proceeding slowly since the FDA approval of levodopa in 1970. Levodopa remains the most effective drug therapy for the motor symptoms of PD, despite its long-term complications [82,83,84]. 

The pathological hallmark of PD is the presence of Lewy bodies within dopaminergic neurons in the brains of affected patients, and misfolded α-syn is known to be the principal component of Lewy bodies [85,86,87,88]. Although the formation of amyloid fibrils by α-syn aggregation plays a central role in the pathogenesis of PD, it has recently been shown that the formation of Lewy bodies—rather than fibrils—is one of the major drivers of neurodegeneration by disrupting cellular functions and inducing synaptic dysfunctions, as well as mitochondrial damage and deficits [89].

Several studies have shown that α-syn oligomers are the primary cause of neurotoxicity and play a critical role in PD, similar to that of Aβ oligomers in AD [90]. Emerging evidence suggests that small soluble α-syn oligomers are the most toxic species among the forms of α-syn aggregates and that size and topological structural properties are crucial factors for oligomer-mediated toxicity, involving the interaction with either neurons or glial cells [91]. 

### 2.3. Neurodegenerative Drug Discovery

Most NDs, including AD, PD, HD, and ALS, remain essentially incurable to date. As mentioned before, current therapy merely alleviates the symptoms but cannot stop the progress of the disease, highlighting the urgent need for more effective therapeutic strategies [4,5]. Barriers to the development of new drugs for NDs include an incomplete understanding of the biology of these multifactorial disorders, the presence of a blood-brain barrier (BBB) that restricts the flow of molecules to the brain, and a lack of clinically relevant animal models on which to test new drugs [92].

Although NDs are characterized pathologically by the aggregation of disease-specific misfolded proteins and changes in cellular stress responses, researchers remain focused almost exclusively on reducing the misfolded protein load (particularly that of Aβ in drug development for AD); however, outcomes have been disappointing [50,93].

Given the low success rate of ND drug discovery, a paradigm shift for innovative drug development strategies was required [94]. New treatments have been proposed, including ones that target various generic stress responses and preventative measures targeting the original misfolded protein triggers, their toxicity, and the spread of aggregates, which may hold promise for the future management of these diseases [93].

Ultimately, the drug discovery paradigm of NDs has gradually shifted from the design of selective drugs targeting a single pathophysiological pathway toward the development of multitarget agents directed at complex pathophysiological pathways of the diseases [44,45,46,47]. Indeed, the complexity and multiple etiologies of NDs make it challenging to obtain desirable therapeutic effects using single-target drugs. Therefore, the use of multitarget directed ligands has emerged in recent years as a powerful strategy to develop potential therapeutics for NDs [95,96,97]. NPs with multiple biological activities that can affect the pathophysiological changes in the brain that contribute to ND development and progression are of particular interest in anti-neurodegenerative drug discovery [98]. 

### 2.4. Natural Products against Neurodegeneration 

The use of phytotherapeutics continues to expand around the world, with many people now resorting to phytomedicine to treat and prevent a wide array of pathologies [99]. Many medicinal plants and their NPs have been reported as being able to alleviate the symptoms of NDs, including AD and PD [100,101,102,103,104,105]. Historically, NPs that are the most important sources of drugs may also hold promise for treating NDs [106,107].

A number of medicinal plants contain active components that are known to possess antioxidant action [108,109]. Abundant data in the literature suggests that dietary NPs found in fruits and vegetables are powerful antioxidants that offer health benefits against several oxidative stress-induced NDs, including AD [110,111]. Most of these NPs have remarkable antioxidant properties and act mainly by scavenging free radical species [109,112].

Since oxidative stress has long been associated with neurodegeneration, there has been a significant increase of interest in finding natural and synthetic compounds with antioxidant and anti-inflammatory effects as promising drug candidates for treating NDs [113]. In recent years, several natural antioxidants have been exploited for their actual or supposed beneficial effect against oxidative stress, including flavonoids and polyphenols [114,115]. Likewise, plant-derived antioxidant polyphenols have come under the research spotlight due to their potential to prevent oxidative stress [109,112].

Several dietary phytochemicals with known antioxidant properties and anti-amyloidogenic effects have been investigated for their potential beneficial effects, including curcumin, resveratrol, and green tea catechins like EGCG [111]. In particular, green tea catechins have been highlighted as having potential protective effects against NDs due to their diverse array of physiological actions, which include potent antioxidant effects [116,117]. 

Notably, because of their broad spectrum of pharmacological and biological activities, NPs are considered promising alternatives for treating neurodegeneration as they might play a role in ND drug development and discovery [118,119,120,121]. NPs remain a promising pool for discovering scaffolds with high structural diversity and various bioactivities that can be directly developed or used as starting points for optimization into novel drugs [122]. Many of these NPs are known as multi-targeted compounds as they alter multiple pathways at the molecular level, making them ideal therapeutic options for multifactorial and complex diseases such as NDs [7].

NPs have, therefore, emerged as potential multi-targeted agents for treating NDs. The major mechanisms through which NPs exert their neuroprotective effects include antioxidant, anti-inflammatory, antithrombotic, antiapoptotic, and neurotrophic activities, as well as acetylcholinesterase and monoamine oxidase inhibition [123]. Among neuroprotective NPs, phenolic molecules are of particular interest since most can target both amyloid aggregation and oxidative stress, as confirmed by numerous studies with phenolic compounds such as EGCG, curcumin, resveratrol, quercetin, and oleuropein [123,124,125,126]. Evidence also exists that some of these NPs suppress the neurotoxicity of the most toxic oligomer species [127,128,129,130,131].

Finally, targeting protein misfolding with different NPs has been recognized as one of the most promising therapeutic strategies against NDs since many NDs involve the misfolding and aggregation of specific proteins into abnormal, toxic species [125,132]. Therefore, the use of small molecules to stop, slow, or reverse the protein misfolding and aggregation process may be a valuable approach to reduce neurodegeneration [17,20]. Concerning the molecular mechanisms by which NPs target misfolded proteins, it is likely that they interfere with electrostatic and hydrophobic interactions which stabilize β-sheets due to the establishment of intermolecular interactions with sidechain or backbone residues of the protein. These intermolecular interactions might be covalent and non-covalent such as hydrogen bonding, π-π interactions, or charge-charge interactions [132]. 

## 3. Protein Misfolding in Neurodegenerative Diseases

**Figure 2:**
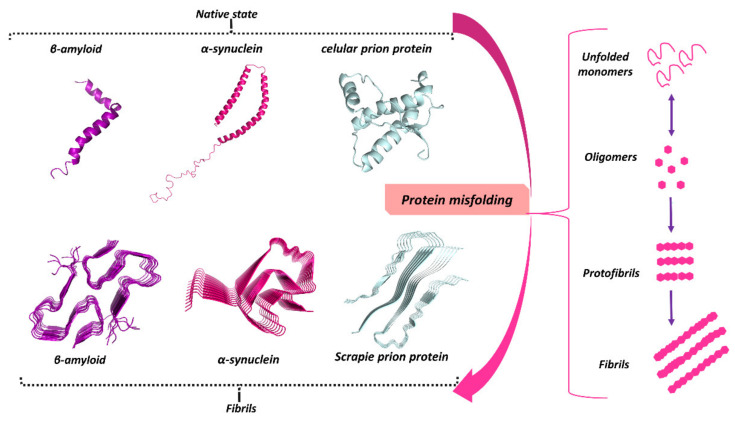
Schematic representation showing that neurodegenerative diseases (NDs) share their origin in protein misfolding followed by the formation of β-sheet-enriched structures. These protein misfolding events are the molecular alterations that trigger several NDs. 3D structures of monomers and fibrils retrieved in the protein data bank (PDB). Aβ monomer (PDB code: 1IYT); α-syn monomer (PDB code: 1XQ8); murine cellular prion protein (PDB code: 1AG2); Aβ fibril (PDB code: 5KK3); α-syn fibril (PDB code: 2N0A); prion protein fibril (PDB code: 6UUR). The protein structures here shown were obtained from recombinantly expressed proteins in vitro, except for Aβ monomer, a chemically synthesized peptide.

The conversion of proteins from their native state to misfolded aggregates is associated with and thought to be the cause of some NDs, including AD and PD [133]. The misfolding, aggregation, and deposition of specific proteins is the key characteristic of most progressive NDs [21,134]. Aβ, tau, α-syn, TDP-43, huntingtin, or the prion protein (PrP) are just a few examples of disease-specific proteins that can aggregate and contribute to the pathogenesis of NDs (Figure 2). The misfolded protein aggregates cause cellular toxicity and eventually lead to cell death in neurodegenerative pathologies. In all cases, the aggregation process plays a key role in the disease progression either due to a loss of protein function (as a result of the aggregation itself) or to the toxicity of soluble aggregates [17,22].

Given that NDs originate through a common pathway of aggregation, with aggregates sharing a similar structure, it is not surprising that these aggregates cause cellular damage through similar mechanisms. However, key differences among misfolded proteins occur due to the location of the aggregates, whether intra- or extracellular and their concentration, which depends on several factors, including the stability of the fibrils [93]. It is well known that the misfolding, aggregation, and accumulation of proteins culminate in damage to the neurons in which the proteins accumulate, causing the neurodegeneration process [135]. A particular protein can fold into a stable alternative conformation, which in most cases results in its aggregation and accumulation in tissues as fibrillar deposits [136]. 

**Figure 3:**
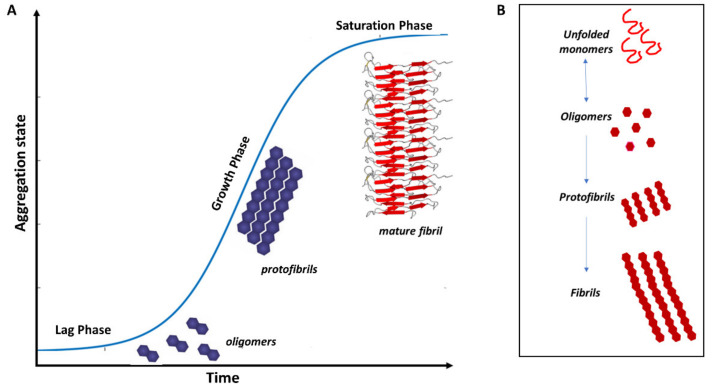
Schematic representation of the amyloid aggregation process. (**A**) Aggregation kinetics of misfolded proteins: the sigmoidal curve represents the temporal evolution of a fluorescent signal. (**B**) Under pathological conditions, natively unfolded monomers are able to self-aggregate in pathological oligomers (primary nucleation). Thus, oligomers can be extended into protofibrils (secondary nucleation) and mature fibrils.

Increasing evidence shows that the assembly of amyloid fibrils is accompanied by conformational changes in the aggregating proteins. The ‘natively unfolded’ polypeptides Aβ and α-syn, for example, have a primarily random-coil structure in their soluble, native state [24]. However, in the fibrillogenesis process (Figure 3), their structure converts into a β-sheet conformation, suggesting that β-sheet formation drives the amyloid assembly process. In addition, in vitro experiments suggest that these misfolded proteins readily form cross-β structures with an aggregation kinetics profile that typically displays a sigmoidal curve where the proteins assemble into oligomers (lag phase) prior to fibril elongation (growth phase) and a plateau where the fibrils and free monomers are in equilibrium (saturation phase) [137,138].

Indeed, amyloid fibril formation is a complex, multiphase process consisting of three phases: nucleation or the lag phase, elongation or the growth phase, and a saturation phase. The lag phase starts with monomers undergoing structural rearrangements and self-assembly into dimers, trimers, and/or oligomers. In the growth phase, the oligomeric nucleus acts as a template for the monomers in solution and proceeds by fibril elongation, aided by fragmentation, secondary nucleation, and fibril conjoining. Finally, the fibril formation reaches an equilibrium in the saturation phase with mature fibrils and a reduced concentration of the monomeric species [138].

Notably, Stanley Prusiner’s discovery that the PrP can misfold into a pathological conformation that encodes structural information capable of both propagating and inducing severe neuropathology has contributed significantly toward understanding other NDs [139,140]. While there is increasing evidence that other NDs, especially AD (Aβ and tau) and PD (α-syn), exhibit at least some of the same properties as misfolded PrP, many NDs with a protein misfolding component are now referred to as ‘prion-like’ [141,142].

Even though distinct proteins are involved in each ND, the process of protein misfolding and aggregation is strikingly similar. Misfolded proteins are transferred between cells, becoming what is referred to as ‘pathological seeds’ [136]. Experimental studies suggest that these assemblies come from the prion-like seeded aggregation of specific misfolded proteins that upbuild and accumulate to form the intracellular and/or extracellular lesions typical of each disorder [135,143,144]. The prion paradigm has thus emerged as a unifying molecular basis for the pathogenesis of many NDs [145].

The prion paradigm holds that the misfolding and seeded aggregation of certain proteins is a fundamental cause of specific disorders. This discovery has vast implications for understanding the mechanisms involved in the initiation and progression of NDs, as well as for the design of novel treatment and diagnosis strategies. Researchers are now focusing on developing therapies for protein misfolding disorders that employ diverse strategies. These include inhibiting the production of disease-relevant proteins that are prone to misfolding, inhibiting the aggregation of misfolded proteins, removing and preventing the spread of aggregated misfolded proteins, and manipulating cellular systems to mitigate the toxic effects of misfolded proteins [19].

Since protein misfolding and aggregation have been shown to be the leading cause of many NDs, several studies have examined the potential of targeting the fibrillization process of amyloid proteins to combat neurodegeneration. An array of compounds has been identified as potential inhibitors or modulators of protein misfolding and aggregation. Notably, most of the promising molecules that have been identified target misfolded Aβ and α-syn; these molecules appear to bind to oligomers and larger aggregates such as amyloid plaques [146,147]. Such compounds can be categorized into three main types: antibodies, peptide inhibitors, and small molecule inhibitors such as NPs [132]. 

Finally, in view of multiple lines of evidence that support protein misfolding as a common cause and pathological mechanism in NDs, it has been suggested that a common therapy for these incurable disorders might be possible [24]. It is important to note that the potential of EGCG against many NDs reinforces the possibility of developing a common drug therapy for different NDs. In recent decades, the role of natural polyphenol EGCG against protein misfolding and aggregation has been widely studied. A variety of evidence is now available to support the potential of EGCG to inhibit fibrillization and potentially to induce the disassembly of misfolded aggregated proteins (Aβ, tau, and α-syn) [148,149,150,151].

### 3.1. Misfolded Aβ in AD

Aβ is a misfolded peptide involved in AD pathogenesis, and the plaque composed of aggregated Aβ peptide features prominently in AD pathology; thus, most drugs tested for AD over the past two decades have targeted the Aβ peptide [50,66]. The Aβ peptides are 39–42-residue-long peptides found in the senile plaques of AD patients’ brains [61]. These peptides are proteolytic fragments generated by the metabolism of the transmembrane amyloid precursor protein (APP), which then self-aggregate in aqueous solution, going from soluble and mainly unstructured monomers to insoluble ordered fibrils. As soon as Aβ aggregates into fibrils outside the cell, it becomes resistant to proteolytic cleavage [61,152]. 

The cleavage of APP by a complex family of enzymes (γ-secretases and β-secretases) releases Aβ peptides as mainly unstructured monomers [152]. Given the hydrophobicity of the primary structure, Aβ can be divided into four regions: two hydrophobic ones and two hydrophilic ones. The 16 first N-terminal residues constitute a hydrophilic tail, while the two hydrophobic regions are comprised of the central L17–A21 portion and the C-terminus A30–V40/A42, which are separated by the central hydrophilic region E22–G29. The two Aβ hydrophobic regions exhibit a secondary structure propensity for β-structures. These regions transiently adopt β conformations and may then transiently fold into a hairpin. It has been suggested that Aβ monomers in solution adopt a transient hairpin-like conformation, whereas residues D23 to K30 are directly involved in hairpin formation [152].

As mentioned previously, despite the harmful properties of senile plaques, a large body of evidence implicates soluble oligomeric Aβ as the most neurotoxic molecular species [70,153,154]. The misfolding and extracellular aggregation of Aβ peptides have been recognized as the main cause of AD progression, leading to the formation of toxic Aβ oligomers and deposition of β-amyloid plaques in the brain [61]. It has been suggested that oligomeric Aβ species may represent a valid biological target [66,155,156].

### 3.2. Misfolded α-Syn in PD

The α-syn protein is predominantly localized at synaptic sites, where it interacts with many partners such as monoamine transporters, cytoskeletal components, lipid membranes, chaperones, and synaptic vesicle (SV)-associated proteins [157]. The α-syn is monomeric and disordered in its physiological form, though some studies debate whether it adopts a helical tetramer in vivo [158,159,160].

In addition to intracellular aggregation of the misfolded α-syn being linked to PD, it has been implicated in other NDs like AD, multiple system atrophy (MSA), and dementia with Lewy bodies. Furthermore, a disorder group called synucleinopathies is characterized by the accumulation of inclusions rich in the α-syn protein that can appear later in life [161,162]. It is thought that different types of aggregated species (oligomers, protofibrils, fibrils, and others) are formed during the process of α-syn aggregation in these synucleinopathies and that at least some might be neurotoxic and lead to neurodegeneration [163]. Hence, targeting neuronal accumulation of α-syn is appealing as a potential method to halt or delay the progression of PD and other synucleinopathies [164].

The 140-amino acid α-syn is a 14 kDa neuronal protein encoded by the SNCA gene on human chromosome 4 [165]. Its primary amino acid sequence can be divided into three major domains: the N-terminal domain (1–60), the central domain (61–95), and the C-terminal domain (96–140). The N-terminal domain, which contains a multi-repeated consensus sequence (KTKEGV) and has an α-helical propensity, is characterized by an amphipathic lysine-rich amino terminus, which plays a crucial role in modulating its interactions with membranes and a disordered, acidic carboxy-terminal tail. The tail has been implicated in regulating its nuclear localization and interactions with metals, small molecules, and proteins. The central region of α-syn contains a highly hydrophobic motif known as the non-amyloid-β component of AD amyloid plaques (NAC), which is involved in α-syn aggregation when acquiring the β-sheet structure. The NAC region is indispensable for α-syn aggregation. The C-terminal domain is enriched with negatively charged and proline residues, providing flexibility to the polypeptide [166,167].

Since the NAC region of α-syn confers a high propensity for the protein to misfold, it forms β-sheet rich amyloid assemblies, also termed fibrils, under pathological conditions. However, the α-syn assembly into amyloid fibrils is dynamic, and the existence of intermediate oligomeric species has been studied extensively [164]. Similar to oligomeric Aβ, α-syn oligomers cause more neurotoxic effects than larger fibrillar assemblies, inferring that they might, in fact, be the main pathogenic species [168].

The toxic intermediate hypothesis builds on this view, suggesting that toxic oligomers disrupt the integrity of membranes via amyloid pore formation, while the fibrillar end-products are simply a by-product of detoxification. This view is supported by the fact that the fibril-containing Lewy bodies are often found in healthy dopaminergic neurons [169,170,171].

## 4. EGCG for Treating Neurodegenerative Diseases

In light of the major potential of using green tea in ND treatment, green tea catechins have been extensively studied, including in vitro and in vivo studies and clinical trials [16,117]. The therapeutic potential of EGCG, the major bioactive compound of green tea, is now well known in ND research [10]. Over the last 20 years, EGCG has been shown to counteract oxidative stress and improves AD- and PD-like phenotypes in different in vitro and in vivo models (Table 2 and Table 3).

In addition to displaying well-demonstrated neuroprotective effects, EGCG has emerged as a promising modulator of amyloid aggregation that can prevent the toxicity of misfolded protein aggregates in a range of experimental models of NDs. A wealth of evidence is now available to support EGCG as a potent anti-amyloidogenic agent that interacts with a set of amyloidogenic proteins, such as Aβ in the case of AD and α-syn in the case of PD (Table 4).

### 4.1. Evidence from In Vitro Neurotoxicity Models 

In the 1990s, studies with the Aβ-induced neurotoxicity model showed that the presence of Aβ_1-42_ leads to neurotoxicity and increased protein oxidation and, as a result, oxidative stress [220,221,222]. The neurotoxicity of the Aβ protein is mediated through oxygen-free radicals and can be attenuated by antioxidants and free radical scavengers. The attenuation of oxidative stress by antioxidant compounds can, therefore, be a potential therapeutic strategy for treating AD. In the early 2000s, the potent antioxidant properties of the green tea polyphenol EGCG were investigated in an Aβ-induced neurotoxicity model using cultured hippocampal neurons, with the results suggesting that EGCG has protective effects against Aβ-induced neuronal apoptosis from scavenging reactive oxygen species. This was one of the first reports on the benefits of EGCG for preventing AD [172]. 

Subsequently, the molecular mechanism underlying the neuroprotective effect of EGCG in the Aβ-induced neurotoxicity model was investigated with a focus on the cellular metabolism of reduced glutathione with antioxidant properties. The results indicated that EGCG treatment fortified the cellular glutathione pool via elevated expression of γ-glutamylcysteine ligase [176]. 

Oxidative stress has also been shown to induce BACE-1 protein upregulation in neuronal cells, which is the rate-limiting enzyme in APP processing and Aβ generation, as well as being a therapeutic target for AD [223,224]. Although exposure of Aβ_1-42_ to neuronal culture increased BACE-1 protein levels, EGCG treatment significantly attenuated the Aβ-induced production of radical oxygen and β-sheet structure formation [174].

In the early 2010s, it was known that EGCG inhibits Aβ and α-syn fibrillogenesis in cell-free assays; researchers then investigated whether EGCG can remodel insoluble Aβ and α-syn aggregates in a cell model system [148]. This research showed that EGCG can reduce cellular toxicity of mature Aβ and α-syn fibrils by remodeling their structure. The EGCG-mediated remodeling of β-sheet-rich amyloid structures leads to the appearance of smaller amorphous protein aggregates that are nontoxic to mammalian cells [148]. 

In 2017, the employment of α-syn-transduced PC12 cells was performed to investigate the protective effects of EGCG, providing evidence that EGCG can protect these cells against α-syn-induced damage by inhibiting the overexpression and fibrillation of α-syn in the cells [151]. 

A more recent study reported that EGCG can interfere with Cu(II)-induced fibrillation of α-syn and protect cell viability. The researchers demonstrated that EGCG inhibits the generation of Cu(II)-induced reactive oxygen species (ROS), leading to reduced overexpression and fibrillation of α-syn in the cells. Moreover, the combination of Cu and EGCG exhibited better cryoprotection than EGCG alone. Here, it is worth noting that Cu(II) is an oxidant that also accelerates fibrillation and protein aggregation [183].

There are also pieces of evidence of neuroprotective effects of EGCG in neurotoxicity models of PD Parkinsonian neurotoxins include compounds like 6-hydroxydopamine (6-OHDA), 1-methyl-4-phenyl-1,2,3,6-tetrahydropyridine (MPTP), rotenone (ROT), and paraquat. EGCG has demonstrated remarkable neuronal protection against paraquat, 6-OHDA, and MPP+ neurotoxicity, though not against ROT [173,175,178,180,181]. 

In an evaluation of the neuroprotective effects of EGCG on ROT-treated dissociated mesencephalic cultures and organotypic striatal cultures, EGCG partially counteracted the effects of ROT in striatal slice cultures through the reduction of nitric oxide (NO) but did not dissociate cells against ROT toxicity [178]. Although the latter study did not demonstrate that EGCG protects against ROT-induced neurotoxicity in cell models, it was recently reported that EGCG had a neuroprotective effect in vivo on ROT-induced PD models [206,207]. Together, these results from in vitro neurotoxicity models support the notion that EGCG can be used as a neuroprotective agent to treat NDs.

### 4.2. Evidence from Animal Models

Because of its broad spectrum of pharmacological activities, EGCG displayed therapeutic potential on various in vivo models of NDs, including AD, PD, HD, and ALS (Table 2). The therapeutic potential of EGCG in an animal model of AD was first reported in 2005 using Swedish mutant APP-overexpressing mice (Tg APPsw). The researchers demonstrated that intraperitoneal (i.p.) injection (20 mg/kg) of EGCG significantly decreased both Aβ levels and Aβ plaques in the brain. The study further demonstrated that EGCG promoted cleavage in the α-C-terminal fragment of APP and elevated the N-terminal APP cleavage product, soluble APP-α. This suggests that the reduction of cerebral Aβ levels is associated with increased α-secretase cleavage activity [184]. 

Moreover, EGCG similarly reduced Aβ deposition in Tg APPsw when administered orally in drinking water (50 mg/kg), as observed in a study conducted in 2008. The results further indicated that EGCG provides cognitive benefits and modulates tau hyperphosphorylation in these AD transgenic mice [186]. Another study involving Tg APPsw mice focused on the molecular mechanism of neuroprotective action of EGCG. EGCG was found to reduce Aβ-induced neurotoxicity by inhibiting glycogen synthase kinase-3β (GSK-3β) activation and c-Ab/FE65 complex nuclear translocation in these transgenic mice [177]. In addition to transgenic mouse models of human AD pathology, toxin-induced models are also considered to be suitable for exploring the therapeutic treatments of NDs. The neuroprotective effects of EGCG have been reported in toxin-induced AD models such as Aβ-induced, LPS-induced, D-galactose (D-gal)-induced, streptozotocin-induced, and aluminum chloride (AlCl_3_)-induced AD models [13]. 

Two studies from the 2000s and early 2010s used the LPS-induced AD model with orally administered EGCG (1.5 or 3 mg/kg). They demonstrated that EGCG prevents apoptotic cell death by preventing elevated levels of Aβ via the inhibition of β- and γ-secretases. The findings also showed that EGCG prevents memory impairment and amyloidogenesis by inhibiting neuroinflammatory-related cytokines released from astrocytes [187,190].

The oral administration of EGCG (1.5 or 3 mg/kg) has also been shown to protect against Aβ-induced memory and coordination impairment in Aβ-induced AD rats, while intragastrical (i.g.) administration of EGCG (2 or 6 mg/kg) had potent neuroprotective effects on aging mice induced by D-gal, acting via antioxidative and antiapoptotic mechanisms [188,189]. Furthermore, additional evidence from toxin-induced models confirms the potential of EGCG to improve oxidative stress with streptozotocin-induced and AlCl_3_-induced models. These studies showed that EGCG can reduce oxidative stress in peripheral and brain tissue and that it may suppress behavioral changes related to toxin-induced cognitive deficits in these animal models [191,195]. 

The senescence-accelerated mouse prone (SAMP8), a spontaneous animal model of accelerated aging, has also been used in studies with EGCG. This animal model is considered a robust model for studying the pathology of sporadic AD [225]. Studies have demonstrated the ability of EGCG to attenuate cognitive deterioration and memory deficits in these mice via i.g administration of low (5 mg/kg) and high doses (15 mg/kg) [193,194].

EGCG administration was shown to upregulate the anti-apoptotic protein Bcl-2 and amplify the Bcl- 2/Bax ratio and an association was found between EGCG-induced reduction in Aβ accumulation and elevated neprilysin expression (the rate-limiting degradation enzyme of Aβ) [193]. In addition to upregulating neprilysin expression, EGCG was shown to inhibit BACE-1 activity and ameliorate abnormal synaptic protein levels in the frontal cortex and hippocampus in the SAMP8 mice model [194]. 

Returning to the transgenic mice model, in the late 2010s, studies with APP/PS1 mice provide additional evidence for the in vivo neuroprotective properties of EGCG. One study investigated the effect of EGCG on neuronal apoptosis induced by endoplasmic reticulum (ER) stress, revealing that EGCG treatment inhibited ER-stress-induced apoptosis in the cerebral cortex of APP/PS1 mice [196].

It has also been suggested that therapies combining EGCG and ferulic acid in aged APP/PS1 mice confer additional benefits over single treatments in terms of improving behavioral deficits, ameliorating cerebral amyloidosis, and reducing Aβ generation [197]. There is also evidence that dual-drug loaded nanoparticles of EGCG/ascorbic acid enhance the therapeutic efficacy of EGCG in APPswe/PS1 transgenic mice [198]. Recently, a study with a well-established preclinical mixed model of familial AD and type 2 diabetes mellitus (T2DM) using transgenic APP/PS1 mice fed with a high-fat diet revealed that EGCG improves cognitive deficits aggravated by an obesogenic diet through modulation of the unfolded protein response [199]. 

EGCG has also been widely studied in toxin-induced animal models of PD, including the classical 6-OHDA and MPTP, which are the two most extensively used neurotoxins for in vivo PD models [226]. The neuroprotective effect of EGCG on the MPTP-induced PD model was first reported in the early 2000s. Oral administration of EGCG (2 and 10 mg/kg), as well as green tea extract (0.5 and 1 mg/kg) containing high levels of EGCG, clearly alleviated dopamine neuron loss in the substantia nigra and TH protein level depletion [201]. In this model, the toxicity of MPTP is mediated by oxidative stress, especially by NO. Another study tested whether EGCG attenuates MPTP-induced PD in mice by inhibiting neuronal NO synthase (nNOS) expression. The outcomes revealed that both green tea extract and EGCG decreased expressions of nNOS in the substantia nigra [202].

In the 2010s, additional studies using the MPTP-induced PD model confirmed the neuroprotective effect of EGCG against MPTP neurotoxicity [204,205]. EGCG was shown to regulate the iron-export protein ferroportin in substantia nigra, reducing oxidative stress and exerting a neurorescue effect against MPTP-induced functional and neurochemical deficits in mice [204]. 

A 2018 study with an MPTP-induced mouse model of PD focused on the effects of EGCG on the peripheral immune system. The outcomes revealed that EGCG treatment protects dopaminergic neurons from MPTP-induced degeneration, restoring the movement behavior of these mice. In addition, EGCG inhibited the expression of neuroinflammatory cytokines and reversed the T cell dysfunction in the peripheral immune system of MPTP mice [205].

An in vivo study with a 6-OHDA-induced PD rat model showed that EGCG administered by gavage (10 mg/kg) reverts the striatal oxidative stress and immunohistochemistry alterations. Furthermore, EGCG treatment attenuated the behavioral changes, indicating neuroprotection manifested as decreased rotational behavior, increased locomotor activity, anti-depressive effects, and improvement of cognitive dysfunction [203].

Recently, another study with a PD rat model focused on the effects of EGCG on ROT-induced motor and neurochemical dysfunctions. This study demonstrated that the possible neuroprotective effects of EGCG (100 or 300 mg/kg i.p.) against ROT-induced motor and neurochemical dysfunctions in rats are associated with its antioxidant effects, prevention of mitochondrial dysfunction, prevention of neurochemical deficiency, anti-neuroinflammatory effects, and anti-apoptotic effects [207].

EGCG supplementation was recently shown to result in profound changes in gut microbial compositions in an invertebrate PD model, restoring the abundance of a set of bacteria [206]. The study with PTEN induced putative kinase 1 (PINK1) mutant flies showed that EGCG ameliorates neuronal and behavioral defects by remodeling gut microbiota [206]. In PD animal models, gut microbiota regulates motor deficits and neuroinflammation [227]. Interestingly, emerging evidence suggests that EGCG remodels the architecture of human gut microbiota [228,229]. However, further studies are necessary to state that modulation of gut microbiota is related to EGCG’s mode of action. 

Researchers are now convinced that gut bacteria may be involved in many NDs, and remodeling the gut microbiota to maintain their balance might be a novel therapeutic strategy [230,231]. Indeed, the most recent drug approved for AD was sodium oligomannate, whose mode of action is related to gut microbiota and neuroinflammation, highlighting the importance of the gut microbiome as a potential drug target for AD [53].

The wealth of evidence from studies with animal AD and PD models suggests that EGCG may be useful for treating AD and PD. However, the available evidence of EGCG neuroprotective effects is not just limited to studies with AD and PD animal models. EGCG has also demonstrated in vivo potential in preventing other NDs in studies published since the 2000s, including transgenic mouse models of ALS and FAP, a *Drosophila* model of HD, and a 3-nitropropionic acid-induced rat model of HD [25,208,209,210,211]. Taken together, these preclinical animal studies suggest that EGCG has considerable potential as a drug candidate for neurodegenerative drug discovery.

### 4.3. Evidence from Clinical Trials

The efficacy of EGCG has been demonstrated in preclinical models of NDs in the 2000s and 2010s; thus, it was approved for a clinical trial in 2019. To this end, a randomized, double-blind, placebo-controlled parallel-group phase III study was conducted in patients with multiple system atrophy (MSA), a rare ND characterized by aggregation of α-syn in oligodendrocytes and neurons, which shares neuropathological features with PD. However, this trial found that supplementation of EGCG did not affect MSA progression. The same study found that although EGCG was generally well tolerated, it was associated with hepatotoxic effects in some patients, thus doses of more than 1200 mg should not be used [232]. There are also reports that phase II clinical trials for AD (NCT00951834) and HD (NCT01357681) have been completed. 

Given the role of oxidative stress in the pathogenesis of NDs, antioxidant therapy for NDs has received considerable attention in recent years as a promising approach to delay or slow oxidative stress-induced neurodegeneration. Many antioxidants, including EGCG, have undergone clinical studies in recent years. Nevertheless, there are barriers to taking antioxidants from the preclinical stage to clinical settings, which are mainly related to pharmacokinetic and pharmacodynamic constraints. Hence, despite promising outcomes in in vitro and in vivo assays, few to no positive results have been attained to date in clinical trials for any of the antioxidants investigated [233].

Researchers have assumed that the low bioavailability of EGCG was an important factor behind these inconsistencies. Indeed, it has been suggested that the therapeutic potential of EGCG is limited by its poor systemic absorption following oral administration, including low absorption, poor pharmacokinetics and bioavailability, scarce biodistribution, first-pass metabolism, trivial penetration, and low accumulation in the related tissues of the body, or low targeting efficacy. Furthermore, EGCG is unstable under physiologic conditions, and it can be rapidly degraded or metabolized due to interactions with the hydroxyl groups on the phenol rings. Intravenous administration of EGCG results in partial degradation before it reaches the target tissues [234].

## 5. EGCG Targeting Misfolded Aggregates in AD and PD

The in vitro effects of NPs that target protein misfolding have been widely studied by numerous independent research groups, combining biochemical and biophysical characterizations with imaging techniques and cell viability assays. These included transmission electron microscopy (TEM), atomic force microscopy (AFM), thioflavin (ThT) binding assay, dynamic light scattering (DLS), circular dichroism (CD) spectroscopy, Fourier-transform infrared (FTIR) spectroscopy, small-angle x-ray scattering (SAXS), and nuclear magnetic resonance (NMR) [125].

Multiple studies have shown the effectiveness of EGCG at inhibiting Aβ and α-syn aggregation and other proteins (Table 4). EGCG was first reported in 2006 by Dagmar E. Ehrnhoefer et al. as a modulator of early events in huntingtin misfolding, which changes the conformation of the huntingtin and prevents the formation of destructive protein-protein interactions [25].

Next, Ehrnhoefer et al. turned to investigate whether EGCG has the same effect in the fibrillogenesis of other aggregation-prone ‘natively unfolded’ polypeptides such as Aβ and α-syn. They were able to show that EGCG effectively inhibited the fibrillogenesis of both α-syn and Aβ by directly binding to the natively unfolded polypeptides, which can also prevent their conversion into toxic, on-pathway aggregation intermediates. This suggests that EGCG recognizes unfolded polypeptides and directly binds to the main chain common to all proteins [26]. 

This discovery suggests that EGCG could potentially be a candidate for developing a common pharmacological therapy for NDs and protein misfolding and amyloid diseases in general. Since these findings became known, many studies postulated that EGCG can reduce the neurotoxicity of misfolded proteins related to numerous NDs. Most studies in this area have been focused on Aβ and α-syn aggregation and toxicity (Table 4).

In the early 2010s, after findings demonstrated that EGCG redirects amyloidogenic polypeptides into unstructured, off-pathway oligomers, researchers investigated whether EGCG disassembles preformed amyloid fibrils. The results revealed that the EGCG treatment of amyloid fibrils does not reverse the amyloid formation process but rather directly converts fibrillar species into benign protein aggregates, suggesting that EGCG is a potent remodeling agent of mature amyloid fibrils [148].

Researchers found that EGCG binds to preformed Aβ and α-syn amyloid fibrils and oligomers and directly alters their morphology, as confirmed by biochemical, biophysical, and cell-based assays, providing experimental evidence that EGCG directly binds to β-sheet-rich aggregates, mediating a conformational change without disassembling them into monomers or small diffusible oligomers [148]. It was proposed that a compound-mediated reorientation of bonds between ordered protein molecules in the polymer might be responsible for amyloid remodeling and the appearance of unordered, amorphous protein aggregates.

On the other hand, another study has suggested that EGCG can robustly disaggregate pre-formed oligomers and has a potent dose-dependent inhibitory activity on α-syn aggregation. This study used confocal single-molecule fluorescence spectroscopy to characterize the effects on α-syn oligomer formation of 14 phenolic compounds and black tea extract, including EGCG. The structure-activity analysis concluded that the presence of three vicinal groups on a single phenyl ring, which has been observed in compounds as EGCG, can be an important molecular feature for more effective in inhibiting and destabilizing self-assembly by α-syn [212].

Concerning the chemical mechanisms of EGCG–Aβ interactions, one study showed that EGCG interferes with the aromatic hydrophobic core of Aβ, forming nontoxic Aβ oligomers. Using magic angle spinning solid-state NMR investigations, researchers demonstrated that the nontoxic aggregates formed in the presence of EGCG are well structured, with a characteristic hairpin structure commonly observed for Aβ fibrils and oligomers [128].

A study aimed at understanding the molecular mechanism by which EGCG remodels mature amyloid fibrils was published in 2013 and showed that EGCG amyloid remodeling activity in vitro is dependent on auto-oxidation of the EGCG [213]. EGCG can self-oxidize, affording a complex mixture of monomeric and polymeric EGCG-based quinones, and auto-oxidation has been considered one of the major reactions causing the in vitro instability of EGCG [235]. 

Researchers have suggested that the molecular mechanism by which EGCG remodels mature amyloid fibrils may be partially due to hydrophobic binding and Schiff base formation with lysine residues of proteins. It was hypothesized that aberrant post-translational modifications mediated by EGCG treatment could occur concomitantly with the hydrophobic remodeling process that seems to be the main driver for EGCG amyloid remodeling. Finally, the primary hydrophobic binding mechanism suggested for amyloid remodeling by EGCG and, more likely, its oxidation products may also explain the ability of EGCG to prevent the fibrillar aggregation of monomeric amyloidogenic proteins, perhaps by binding to and making oligomeric seeds less kinetically competent [213].

Additionally, another study from 2013 noted that oxidized EGCG can be useful to fabricate drug delivery microparticles from naturally reproducible and edible green tea [236]. More recently, it was demonstrated that green tea polyphenol microparticles based on the oxidative coupling of EGCG inhibit amyloid aggregation/cytotoxicity of the protein α-syn and serve as a platform for drug delivery [219]. The study proposed using EGCG microparticles as a possible bifunctional strategy, blocking amyloidogenesis directly and carrying a molecule that can act synergistically to potentiate the anti-amyloidogenic effect [219].

In 2014, a study that combined NMR spectroscopy, TEM, and CD characterized the interaction of green tea catechins (epicatechin, epigallocatechin, epicatechin gallate, and EGCG) with toxic oligomers in detail. The flavan-3-ol unit of catechins was shown to be essential for EGCG–Aβ interactions. In addition, similar experiments still demonstrated that the same flavan-3-ol unit is also essential for the interaction with oligomers of PrP [214].

The effect of EGCG on tau aggregation has also been reported. This effect was characterized in a study conducted in 2015, which combined cell-free aggregation and a cell-based assay, and found that EGCG could be a potent inhibitor of tau aggregation and toxicity, preventing the aggregation of tau protein into toxic oligomers at substoichiometric ratios [150]. More recently, a study with various biophysical and biochemical analyses suggested a possible dual effect of EGCG on aggregation inhibition and disassembly of full-length tau and their binding affinity [28]. 

Returning to studies of α-syn oligomers, it has been proposed that the EGCG’s mechanism of action to inhibit the formation and block the toxicity of oligomers is related to EGCG affecting the degree of binding of α-syn oligomers to membranes, resulting in the reduction (but not complete elimination) of the oligomer’s affinity for cell membranes. According to in vitro assays, this does not affect oligomer size distribution or secondary structure. The data demonstrated that EGCG immobilizes the C-terminal region and moderately reduces the binding degree of oligomers to membranes [149]. 

**Figure 4:**
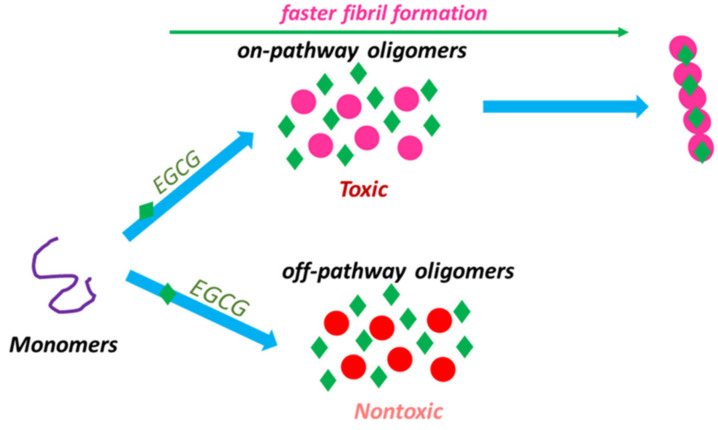
Schematic illustration showing the effect of EGCG on α-syn aggregation. EGCG induces the formation of two distinctive types of α-syn oligomers. The accelerated amyloid fibril formation is also observed in the presence of EGCG.

As a matter of fact, it has been recognized that EGCG does not inhibit α-syn fibril formation but rather exerts protection against α-syn cytotoxicity, both by enriching the population of nontoxic off-pathway oligomers as well as by promoting the conversion of toxic oligomers into the less toxic fibril (Figure 4). This might be attributed to EGCG binding to on-pathway oligomers and accelerating their conversion to fibrils so that the effective population of toxic oligomeric intermediates can be reduced [131]. 

In this regard, a 2017 study by Yang et al. provided convincing evidence that EGCG does not inhibit α-syn fibrillation but actually facilitates it, contradicting previous reports that EGCG acts as a remodeling agent favoring the production of small amorphous protein aggregates from mature fibrils [26,131]. A sample of co-incubated mixture of α-syn with EGCG completely diminished the fluorescence signal in a ThT binding assay, indicating a possible full inhibition of amyloid formation. Morphologically altered fibrils which appeared slightly curly compared to the straight fibrils obtained in the same fibrillation conditions but in the absence of EGCG were observed. The authors thus suggested that the ThT binding assay is inappropriate to evaluate the ‘inhibitory’ effect of EGCG on α-syn fibrillation [131].

In fact, although ThT is a gold standard probe to detect amyloid fibrils in vitro, it has been recommended caution to test polyphenols using the ThT binding assay since some critical limitations have been reported such as background signal and competitive binding with the extrinsic probe. This method needs to be validated by other techniques to confirm that the significant decrease in ThT fluorescence observed in the presence of the evaluated compound is related to an inhibitory effect on amyloid fibrillation or rather by compound displacement by ThT [237,238,239,240].

It should be mentioned that the reduction in ThT emission in the presence of EGCG might result from the competition of this compound with ThT for amyloid fibrils binding sites [237,238]. Recently, pentameric thiophene fluorescence was proposed as an alternative probe to monitor the aggregation kinetics in the presence of EGCG [240]. According to the study by Yang et al., it is pertinent to consider that EGCG induces two types of α-syn oligomers. The TEM and AFM findings revealed that EGCG-treated α-syn not only produces the off-pathway compact oligomers but also produced amyloid fibrils formed by the conversion of on-pathway toxic oligomers into fibrils. This supports the hypothesis that EGCG acts to facilitate the conversion of on-pathway oligomers into amyloid fibrils, suppressing this population of toxic oligomers [131].

In a recent study, researchers confirmed that EGCG can accelerate α-syn amyloid fibril formation by facilitating its heterogeneous primary nucleation. This work showed that the aggregation conditions define whether EGCG is an inhibitor or enhancer of α-syn amyloid fibril formation, since the inhibitory action is not robust against various physiologically relevant changes in experimental conditions [241].

Taken together, these last findings suggest that EGCG can exert its neuroprotective effect against α-syn-induced cytotoxicity by modifying the aggregation pathway toward the formation of nontoxic aggregates and ameliorating oligomer-induced toxicity, possibly through reducing the extent of cell membrane permeabilization induced by toxic aggregates [131,149].

In another study published in 2017, the effects of EGCG on the fibrillation and disaggregation of α-syn at a molecular level were assessed by using chemical, biochemical, and cell-based methods like ThT binding assay, NMR, microscopy, and MTT. These studies indicated that EGCG binding to Leu, His, Phe, and Tyr aminoacid residues inhibits the conformational transition of α-syn to β-sheet-enriched conformers and also disaggregates the amyloid fibrils of α-syn in a dose-dependent manner [151].

Additionally, in 2017, a study compared the effect of EGCG on α-syn aggregation to other three inhibitors (dopamine, amphotericin-B, and quinacrine dihydrochloride). The experiment conducted in this study revealed that, at high concentrations, EGCG slows down fibrillization kinetics. EGCG was also able to reduce the toxicity of α-syn aggregates in a concentration-dependent manner. The morphological study of nontoxic aggregates formed in the presence of EGCG showed a smaller fibrillar size. Researchers proposed that the decrease in cytotoxicity of α-syn aggregates in the presence of a higher concentration of EGCG can be attributed to its ability to reduce the exposure of a hydrophobic surface, as shown in 8-anilino-1-naphthalenesulfonic acid (ANS) binding studies [215].

Interestingly, the ANS binding assay has already provided valuable insights into the role of exposure of hydrophobic surfaces as a result of the aggregation of misfolded species, which is a crucial and common feature of these pathogenic species [242]. It is now known that the exposure of hydrophobic groups on the oligomer surface appears to be a major determinant of oligomer-mediated toxicity. A range of proteins, oligomeric species of similar sizes and morphologies but with very different toxicities, have been isolated and shown to differ in their solvent-exposed hydrophobicity [243].

There is also strong evidence that Aβ toxicity is regulated by the solvent exposure of hydrophobic surfaces. Meanwhile, the exposure of hydrophobic surfaces is the decisive factor for toxicity rather than the presence of β-sheets. Multiple lines of evidence suggest that exposure to these toxic surfaces facilitates interactions with multiple cellular components, including membranes, which underlie key pathogenic steps in AD progression [244,245].

Since the exposure of hydrophobic surfaces seems to remodel misfolded protein aggregation, recent studies reported that the mechanism of action of EGCG against protein misfolding is directly related to the regulation of the solvent exposure of hydrophobic surfaces. EGCG, therefore, reduces the toxicity of misfolded aggregates by binding to preformed oligomers and fibrils and altering their hydrophobic surface exposure [216,217].

Ahmed et al., in 2017, elucidated how EGCG remodels Aβ oligomers using ^15^N and ^1^H dark-state exchange saturation transfer (DEST), relaxation, and chemical shift projection NMR analyses with fluorescence, DLS, and electron microscopy. The experimental findings suggested that the mechanism of amyloid inhibition of EGCG is driven by the preferential binding to Aβ oligomers [216]. The solution NMR studies concluded that EGCG can bind weakly to Aβ monomers while it displays a higher affinity toward oligomers. It is speculated that the Aβ_40_ oligomers become less exposed to solvent upon binding to EGCG, and the β-regions, which are involved in direct monomer−protofibril contacts in the absence of EGCG, undergo a direct-to-tethered contact shift [216]. 

To gain further insight on the structural determinants of Aβ toxicity, Ahmed et al. investigated Aβ oligomers with different toxicity degrees generated in the absence or presence of a catechin library that included EGCG and six EGCG analogs. By combining cell toxicity assays, electron microscopy, NMR spectroscopy, DLS, wide-angle X-ray diffraction (WAXD), and fluorescence assays, the comparative analysis identified a cluster of key toxicity determinants and the associated mechanism of action, which includes exposure of a hydrophobic surface spanning residues 17–28 and the concurrent shielding of the highly charged N-terminus [217].

The study findings indicated that toxic Aβ assemblies (Aβn) exhibit solvent-exposed hydrophobic sites accessible to ANS binding, while EGCG-remodeled Aβ aggregates are less toxic and have fewer exposed hydrophobic sites. These exposed hydrophobic surfaces facilitate the colocalization, interaction, and subsequent insertion of Aβ assemblies into the membrane. These findings indicate that Aβn toxicity is regulated by the solvent exposure of hydrophobic surfaces [217].

Up to this point of our discussion, all the in vitro evidence strongly suggests that EGCG targets protein misfolding, indicating a potential therapeutic for NDs. However, it is important to recognize that all cited studies have analyzed amyloid fibrils and other aggregates that are formed from chemically synthetic or recombinantly expressed peptides or proteins in vitro, and in vitro amyloid fibril structures may differ profoundly from ex vivo amyloid fibril structures [246,247]. It is unclear whether the assays using protein structures that may not represent the in vivo amyloid structure can truly explain how EGCG acts against protein misfolding in vivo.

## 6. Conclusions 

A considerable body of evidence that supports the use of EGCG for ND therapy has become available over the past two decades. The potential effects of EGCG in NDs are now well described and proven by a range of experimental in vitro and in vivo assays discussed in this review. However, despite extensive evidence from in vitro and in vivo models of AD, PD, and other NDs suggesting that EGCG is a promising drug candidate for ND treatment, clinical evidence on its anti-neurodegenerative effects does not yet exist. In a clinical trial phase III with MSA patients, no effect on disease progression was found, while some patients showed hepatotoxic effects [232].

Some authors have suggested that the inconsistency between the evidence from preclinical stages and clinical trials in humans might be attributed to catechins, like EGCG having poor pharmacokinetic properties and bioavailability, limiting their effectiveness as drug leads. However, this could be improved through new techniques such as nanoparticle-based delivery systems, structurally modified molecules of catechins, or co-administration with other drugs or bioactive compounds [234]. 

The ability of a small molecule to reduce the toxicity of oligomeric species has been shown to represent a promising therapeutic strategy against NDs. EGCG has been shown to significantly reduce the cell toxicity of several amyloid aggregates by binding to preformed oligomers and fibrils and altering their hydrophobic surface exposure. These findings suggest that the use of EGCG could, therefore, be viewed as common drug therapy for many NDs targeting protein misfolding, a common feature of numerous NDs.

It is also important to note that the studies of the mechanisms by which EGCG combats neurodegeneration have already provided significant advances in our knowledge of the mechanisms of protein misfolding toxicity. Furthermore, EGCG and other green tea catechins have been recognized as valuable tools for identifying the drivers of amyloid aggregation and developing other aggregation modulators through structural mimicry [16]. 

Notably, a deeper understanding of the molecular mechanisms of the interactions between small molecules and a misfolded protein may have a prominent role in rational drug design and the development of new therapeutic strategies for NDs. The ultimate message that emerges is that, although current EGCG research has limited impact on clinical practice, it has provided strong evidence and testable hypotheses to contribute to clinical advances and neurodegenerative disease drug discovery.

## Figures and Tables

**Table 1:** Protein misfolding disorders affecting the nervous system [37].

Disease	Misfolded Protein(s)	Cell Types Primarily Affected	Clinical Feature(s)
Alzheimer’s disease (AD)	Aβ, tau	Hippocampal neurons	Dementia
Parkinson’s disease (PD)	α-syn	Substantia nigra Dopaminergic neuron	Parkinsonism
Multiple system atrophy (MSA)	α-syn	Basal ganglia and/or cerebellar oligodendrocytes	Parkinsonism and/or ataxia
Dementia with Lewy bodies (DLB)	α-syn	Cortical and/or hippocampal and/or striatal neurons	Dementia and/or parkinsonism
Huntington disease (HD)	huntingtin	Striatal neurons	Dementia
Spinocerebellar ataxia	Ataxin	Cerebellar neurons	Cerebellar ataxia
Amyotrophic lateral sclerosis (ALS)	Ataxin, FUS, TDP43, C9orf72 or superoxide dismutase 1 (SOD1)	Motor neurons	Muscular atrophy
Frontotemporal dementia	FUS, TDP43, C9orf72 or SOD1	Cortical neurons	Dementia
Gerstmann–Sträussler– Scheinker syndrome (GSS)	Prion protein	Cerebellar neurons	Ataxia
Fatal familial insomnia	Prion protein	Thalamic neurons	Insomnia
Creutzfeldt–Jakob disease (CJD)	Prion protein	Cortical neurons	Dementia

**Table 2:** Selected evidence of EGCG effectiveness on in vitro neurotoxicity models.

Experimental Models	Cell Line	Outcomes
Aβ-induced neurotoxicity model	Primary culture	Elevates cell survival and decreases the levels of malondialdehyde (MDA) and caspase activity [172]
Paraquat-induced PD model	PC12 cells	Protects against paraquat-induced apoptosis via modulating mitochondrial function [173]
Aβ_1-42_-exposure neuronal cells	Primary culture	Suppresses Aβ-induced BACE-1 upregulation [174]
6-OHDA-induced PD model	SH-SY5Y cells	Protects against cell death through STAT3 activation [175]
Aβ-induced oxidative and nitrosative cell death	BV2 microglia	Fortifies cellular antioxidant glutathione pool via elevated expression of γ-glutamylcysteine ligase [176]
Human neuronal cells	MC65 cells (overexpressing APP)	Suppresses Aβ -induced neurotoxicity by inhibiting c-Abl/FE65 nuclear translocation and GSK3β activation [177]
ROT-injured murine brain cultures	Primary mesencephalic cell cultures	No influence on the survival of dopaminergic neurons in mesencephalic cultures [178]
DDT-induced cell death	SH-SY5Y cells	Activates endogenous neuroprotective mechanism(s) that can protect against cell death [179]
Fibril-induced neurotoxicity	HEK-293 cells(overexpressing α-syn)7PA2 cells(overexpressing APP)PC12 cells	Remodels mature α-syn and amyloid-β fibrils and reduces cellular toxicity [148]
MPP+-induced PD model	PC12 cells	Suppresses oxidative stress via the SIRT1/PGC-1α signaling pathway [180]
6-OHDA-induced PD model	N27 cells	Pretreatment with EGCG protected against neurotoxicity by regulating genes and proteins involved in brain iron homeostasis, especially modulating hepcidin levels [181]
Microglia-mediated neuroinflammation	EOC 13.31	Attenuates Aβ-induced inflammation and neurotoxicity [182]
α-syn induced neurotoxicity	α-syn transduced-PC12 cells	Protects cells against α-syn-induced damage by inhibiting the overexpression and fibrillation of α-syn in the cells [151]
Cu(II)-mediated toxicity	α-syn transduced-PC12 cells	Inhibits the overexpression and fibrillation of α-syn and reduces Cu(II)-induced oxidative stress [183]

BACE-1: Beta-secretase-1; BV2: Murine brain microglial cell line; DDT: Dichlorodiphenyltrichloroethane; EOC 13.31: Mouse immortalized microglia cell line; GSK3β: Glycogen synthase kinase 3β; HEK-293: Human kidney (embryonic) cell line; 6-OHDA: 6-hydroxydopamine; 7PA2: CHO cells overexpressing APP; MC65: Human neuroblastoma cell line (MC65) that conditionally expresses a C-terminal derivative of APP; MPP+: 1-methyl-4-phenylpyridinium (ion); N27: Rat mesencephalic dopaminergic neuronal cell line; PD: Parkinson´s disease; PC12: Rat adrenal phaeochromocytoma cell line; ROT: Rotenone; SH-SY5Y: Human neuroblastoma cell line; SIRT1/PGC-1α: Sirtuin 1/peroxisome proliferator-activated receptor gamma coactivator 1-α; STAT3: Signal transducer and activator of transcription-3.

**Table 3:** Main evidence of the neuroprotective effects of EGCG in animal models.

Alzheimer’s Disease		
Experimental Models	Animal Strain	Outcomes
Transgenic mice overproducing Aβ	Tg APPsw (line 2576)	Decreases Aβ levels and plaque associated with promotion of the nonamyloidogenic α-secretase proteolytic pathway [184]
Stereotaxic surgery lesion	Wistar rats	Restores β-amyloid-induced behavioral derangements relating to coordination and memory abilities [185]
Transgenic mice overproducing Aβ	Tg APPsw (line 2576)	Provides cognitive benefit and modulates tau pathology [186]
Transgenic mice overproducing Aβ	Tg APPsw (line 2576)	Inhibits GSK3β activation and c-Abl/Fe65 complex nuclear translocation [177].
LPS-induced AD model	ICR mice	Inhibition of Aβ generation through the inhibition of β- and γ-secretase activity [187]
Aβ-induced AD model	ICR mice	Downregulates β- and γ-secretase activities and eventually decreases toxic Aβ levels in the cortex and hippocampus [188]
D-gal-induced AD model	Kunming mice	Increases the activities of antioxidant enzymes and reduces the activation of caspase-3 [189]
LPS-induced AD model	ICR mice	Prevents activation of astrocytes and elevation of proinflammatory cytokines including TNF-α, as well as the increase of inflammatory proteins such as inducible nitric oxide synthase (iNOS) andcyclooxygenase-2 (COX-2) [190]
Streptozotocin-induced AD model	Wistar rats	Neuroprotective effects through reversion of oxidative stress and decreased acetylcholinesterase activity [191]
Transgenic mice overproducing Aβ	Tg CRND8 mice	Ameliorates some behavioral manifestations and cognitive impairments [192]
Senescence-accelerated mouse (SAM)	SAMP8	Attenuates cognitive deterioration by upregulating neprilysin expression [193]
Senescence-accelerated mouse (SAM)	SAMP8	Oral consumption of EGCG ameliorates memory impairment and reduces the levels of Aβ_1–42_ and BACE-1 [194]
Aluminum-induced AD model	Wistar rats	Oral administration of EGCG nanoparticles attenuates neurobehavioral deficits and Aβ and Tau pathology [195]
Transgenic mice expressing mutant human APP and presenilin 1	APP/PS1 mice	Inhibition of endoplasmic reticulum stress-associated neuronal apoptosis [196]
Transgenic mice expressing mutant human APP and presenilin 1	APP/PS1 mice	Combination of EGCG with ferulic acid improves behavioral deficits, ameliorating cerebral amyloidosis and reducing Aβ generation [197]
Transgenic mice producing abundant Aβ plaques	APPswe/PS1dE9 mice	Oral administration of EGCG/ascorbic acid nanoparticles reduces Aβ plaque burden, Aβ_42_ peptide levels, and neuroinflammation while enhancing synaptogenesis, memory, and the learning process [198]
Transgenic mice producing abundant Aβ plaques fed with a high-fat diet (mixed model of familial AD and T2DM)	APPswe/PS1dE9 mice	Decreases brain Aβ production and plaque burden by increasing the levels of α-secretase and reduces neuroinflammation by the decrease in astrocyte reactivity and toll-like receptor 4 (TLR4) levels [199]
Transgenic mice expressing mutant human APP and presenilin 1	APP/PS1 mice	Reduces Aβ plaques in the brain [200]
**Parkinson’s disease**		
**Experimental models**	**Animal strain**	**Outcomes**
MPTP-induced PD model	C57/BL mice	Alleviates dopamine neuron loss in the substantia nigra and tyrosine hydroxylase (TH) protein level depletion [201]
MPTP-induced PD model	C57B6 mice	Decreases expressions of nitric oxide synthase in the substantia nigra [202]
6-OHDA-induced PD model	Wistar rats	Reverses the striatal oxidative stress and immunohistochemistry alterations [203]
MPTP-induced PD model	C57BL/6J mice	Regulates the iron-export protein ferroportin in substantia nigra, reduces oxidative stress, and exerts a neurorescue effect [204]
MPTP-induced PD model	C57BL/6J mice	May exert neuroprotective effects by modulating peripheral immune response [205]
ROT-induced PD model	*Drosophila melanogaster*	Ameliorates neuronal and behavioral defects by remodeling gut microbiota and turandot M (TotM) expression [206]
ROT-induced PD model	Wistar rats	Reduces NO level and lipid peroxidation production, increases the levels of catecholamines in the striatum, and reduces the levels of neuroinflammatory and apoptotic markers [207]
** Amyotrophic lateral sclerosis **		
**Experimental models**	**Animal strain**	**Outcomes**
Transgenic mice carrying a human SOD1 with a G93A mutation	B6SJL Tg (SOD1-G93A)	Increases the number of motor neurons and reduces the concentration of NF-kB caspase-3 and iNOS [208]
Transgenic mice carrying a human SOD1 with a G93A mutation	B6SJL Tg (SOD1-G93A)	Prolongs symptom onset and life span, preserving more survival signals and attenuating death signals [209]
	**Huntington’s disease**	
**Experimental models**	**Animal strain**	**Outcomes**
Transgenic flies expressing mutant huntingtin fragments with 93 glutamines	*Drosophila melanogaster*	Modulates early events in huntingtin misfolding and reduces toxicity [25]
3-nitropropionic acid induced cognitive dysfunction and glutathione depletion	Wistar rats	Improves memory and restores glutathione system functioning [210]
**Familial amyloidotic polyneuropathy (FAP)**		
**Experimental models**	**Animal strain**	**Outcomes**
Transgenic mice for human TTR	Tg hTTR V30M mice	Decreases non-fibrillar TTR deposition and disaggregation of amyloid deposits [211]

APP/PS1 or PS1dE9: Double transgenic mice expressing a chimeric mouse/human amyloid precursor protein (Mo/HuAPP695swe) and a mutant human presenilin 1 (PS1-dE9); B6SJL Tg: Transgenic mice expressing a G93A mutant form of human SOD1; 6-OHDA: 6-hydroxydopamine; iNOS: Inducible nitric oxide synthase; MPTP: 1-methyl-4-phenyl-1,2,3,6-tetrahydropyridine; ROT: Rotenone; SAMP8: Senescence-accelerated mouse prone; Tg APPsw (line 2576): Overexpresses a mutant form of APP (isoform 695) with the Swedish mutation (KM670/671NL), resulting in elevated levels of Aβ and ultimately amyloid plaques; Tg hTTR V30M mice: Transgenic mice expressing human transthyretin (TTR) with the V30M point mutation.

**Table 4:** Main evidence that EGCG targets toxic misfolded aggregates in neurodegenerative diseases.

Protein	Main Outcome	Experimental Techniques
Huntingtin	Modulates misfolding and oligomerization [25]	Dot-blot and AFM
Aβ_42_ α-syn	Redirects aggregation cascades and thus prevents the formation of toxic, β-sheet–rich aggregation products [26]	ThT fluorescence, TEM, CD, and dot-blot
Aβ_42_ α-syn	Binds to β-sheet-rich aggregates remodeling mature fibrils [148]	ThT fluorescence, TEM, AFM, and CD
α-syn	Inhibits and disaggregates oligomers [212]	Confocal single-particle fluorescence spectroscopy
Aβ_40_	Induces the formation of nontoxic well-structured oligomers [128]	Solid-state NMR and MTT assay
Aβ_40_	The amyloid remodeling activity is dependent on auto-oxidation of the EGCG [213]	ThT fluorescence, congo red assay, EM, AFM, CD, and MTT assay
Aβ_42_ PrP_106–126_	Reduces the number of fibrils [214]	NMR, TEM, and CD
α-syn	Inhibits oligomer toxicity, moderately reduces membrane binding, and immobilizing the oligomer -terminal tail [149]	Calcein release assay, LSCM, NMR, TEM, CD, DLS, SAXS, MTT assay, and ITC
Tau	Prevents aggregation and toxicity [150]	ThT fluorescence, AFM, and MTT assay
α-syn	Inhibits fibrillation and disaggregates amyloid fibrils [174]	ThT fluorescence, CD, NMR, AFM, and TEM
α-syn	Aggregates showed small fibrillar length, and less toxicity correlates with reduction of exposed hydrophobic surface [215]	ThT fluorescence, CD, FTIR, ANS fluorescence, TEM, NMR, SPR, and MTT assay
α-syn	Protects against membrane disruption and cytotoxicity caused by oligomers [131]	ThT fluorescence, tyrosine intrinsic fluorescence, TEM, CD, DLS, FTIR, AFM, and MTT assay
Aβ_40_	Remodels toxic oligomers to nontoxic aggregates [216]	DEST, NMR, ANS fluorescence, DLS, and TEM
Aβ_42_	Remodels soluble Aβ assemblies into less toxic species with less exposed hydrophobic sites [217]	Comparative analysis of N-R2 and DEST NMR combined with WAXD, TEM, DLS, and extrinsic fluorescence
Aβ_42_	Higher EGCG-to-Aβ_42_ ratios promote the rate of aggregation, while lower EGCG-to-Aβ_42_ ratios inhibit the aggregation rate [218]	ThT fluorescence, TEM and EPR
Aβ40	Alleviates aggregation induced by metal ions [200]	ThT fluorescence, TEM, ICP-MS, UV–Vis spectroscopy
Tau	Dual effect on aggregation inhibition and disassembly of full-length Tau [28]	ThT fluorescence, MALDI-TOF analysis, and ITC
α-syn	EGCG microparticles reduce the cytotoxic effects of oligomers; besides, they increase the activity of other antiamyloidogenic compounds when used together [219]	ThT fluorescence, CD, DLS, TEM, and cell viability assay

ANS: 8-anilino-1-naphthalenesulfonic acid ammonium salt; AFM: Atomic force microscopy; CD: Circular dichroism; DEST: ^15^N-dark state exchange saturation transfer; DLS: Dynamic light scattering; EPR: Electron paramagnetic resonance; FTIR: Fourier-transform infrared; MTT: 3-(4,5-dimethylthiazol-2-yl)-2,5-diphenyltetrazolium bromide; ICP-MS: Inductively coupled plasma-mass spectrometry; ITC: Isothermal titration calorimetry; LSCM: Laser scanning confocal microscopy; MALDI-TOF: Matrix-assisted laser desorption ionization time-of-flight mass spectrometry; NMR: Nuclear magnetic resonance; SAXS: Small-angle x-ray scattering; SPR: Surface plasmon resonance; TEM; Transmission electron microscopy; ThT: Thioflavin T; UV–Vis: Ultraviolet-visible; WAXD: Wide-angle X-ray diffraction.
